# Multimorbidity patterns and function among adults in low- and middle-income countries: a scoping review protocol

**DOI:** 10.1186/s13643-022-01996-3

**Published:** 2022-07-07

**Authors:** Karina Berner, Nassib Tawa, Quinette Louw

**Affiliations:** 1 Division of Physiotherapy, Department of Health and Rehabilitation Sciences, Faculty of Medicine and Health Sciences, Stellenbosch University, P.O. Box 241, Cape Town, 8000 South Africa; 2 Centre for Research in Spinal Health and Rehabilitation Medicine, Department of Rehabilitation Sciences, College of Health Sciences, Jomo Kenyatta University of Agriculture and Technology, P. O. Box 62000, Nairobi, 00200 Kenya

**Keywords:** Multimorbidity patterns, Chronic diseases, Functional impairment, Activity limitation, Participation restriction, Physical function, Low-resource settings, LMIC

## Abstract

**Background:**

A fifth of adults in low- and middle-income countries (LMICs) have multimorbid conditions, which are linked to socio-economic deprivation and aging. Multimorbidity is associated with high rates of functional problems and disability, increased healthcare utilization, and lower quality of life. Literature on multimorbidity and associations with function is mostly from high-income countries (HICs) and focused among older adults. Moreover, data regarding disease patterns and their impact on person-centered outcomes are limited. There is a need for research into understanding common patterns of multimorbidity, and their association with functional impairments, particularly in LMICs. Such information may contribute towards evidence-based and context-relevant strategic policy, planning, and delivery models for health and rehabilitation services, which is imperative in attaining Universal Health Coverage (UHC). The planned scoping review aims to provide an overview of the scope and nature of existing literature on multimorbidity patterns and function among adults in LMICs.

**Methods:**

A scoping review will be conducted using a five-step framework and reported according to the PRISMA-ScR guidelines. A comprehensive electronic search of PubMed/MEDLINE, Scopus, EBSCOhost, Scielo, Cochrane and Google Scholar will be conducted and updated from the last pilot search ran in September 2020. Studies of any design will be included if they are reported in English, published (between January 1976 and the last search date) in a peer-reviewed journal, and describe multimorbidity patterns and associations with physical functional impairments, activity limitations or participation restrictions among adults in LMICs. Search results will be independently screened by two reviewers and data extraction will cover study characteristics, participants’ characteristics, multimorbidity measures, patterns analysis, and functional measures. Descriptive statistics and narrative synthesis will be used to synthesize and summarize findings.

**Discussion:**

Patients with multimorbidity have unique and cross-cutting needs, hence the need for integrated and person-centered approaches to policy, planning, and delivery of medical and rehabilitation services. Considering the shift towards UHC and primary healthcare-led management of chronic diseases, the proposed scoping review is timely. Findings will provide insights into the current extent and scope of multimorbidity research, and guide future inquiry in the field.

**Trial registration:**

Open Science Framework (OSF), https://osf.io/gcy7z/

**Supplementary Information:**

The online version contains supplementary material available at 10.1186/s13643-022-01996-3.

## Background

### Multimorbidity in low- and middle-income countries

Many low- and middle-income countries (LMICs) face complex, multiple disease burdens, including chronically managed infectious diseases and rapidly rising non-communicable diseases [1]. Medical advances, increasing population aging, widespread urbanization and food industry globalization add to this unique epidemiologic transition [2]. About a fifth of adults in LMICs suffer from multimorbidity [3] (defined as the co-occurrence of at least two chronic conditions in an individual [4]) and rates may already surpass those in high-income countries (HICs) [5]. Multimorbidity is becoming an increasing public health concern as it is associated with high levels of disability [3, 6], poor quality of life [7], and raises healthcare costs and utilization [8] in already financially constrained public health systems. Additionally, since multimorbidity occurs 10–15 years earlier in LMICs than in less deprived countries [9], these healthcare systems will be strained for longer. Unfortunately, despite recognition of the treatment and rehabilitation challenges associated with addressing multimorbid chronic conditions in a patient, clinical guidelines and health management aspects remain focused on separate singular diseases [10].

Globally, literature on multimorbidity is growing but seems to remain fragmented and poorly understood [4, 5, 11]. Most studies have assessed single diseases or comorbidities in association with an index disease (“comorbidity” is a related yet different clinical entity from “multimorbidity” [4, 12]—see Additional file 1 for a glossary of key terms related to this protocol). Even studies explicitly referring to “multimorbidity” have used different disease count cut-offs, disease combinations, multimorbidity measures and statistical methods for determining patterns. Such methodological differences highlight the current lack of consensus regarding its definition and operationalization [4, 5]. Furthermore, while there is agreement that multimorbidity seems to present in certain disease patterns and leads to functional decline, the available literature is limited, mostly from high-income countries (HICs) and focused among older adults [13]. Such research cannot be generalized to LMICs, where those affected by multimorbidity are relatively younger and the disease patterns are likely different [14].

The lack of agreement on standards for identifying and classifying multimorbidity patterns remains a major challenge when developing clinical guidelines for managing patients with multimorbidity [11]. The healthcare of people with multimorbidity is unique, complex, and different from the highly specialized approaches typically tailored to single diseases [4]. A better understanding of the occurrence and person-centric impact of multimorbidity patterns could thus inform the development, planning, and delivery of targeted and cost-effective interventions for improved patient outcomes.

### Multimorbidity and function

There is much evidence to suggest that multimorbidity leads to declines in physical function, and that a higher number, more severe and certain patterns of co-existing conditions is associated with faster functional deterioration [15, 16]. It has been proposed that the interplay between multimorbidity and function may be two-way. Multimorbidity may lead to disease-disease, drug-drug, or drug-disease interactions, curtailing compensatory mechanisms and resulting in physical and cognitive deterioration. Poor function, on the other hand, may impact the severity and burden of multimorbidity [15]. Limited available research has for example shown that walking speed and handgrip strength are inversely associated with the development and worsening of multimorbidity, with evidence of a dose–response relationship [17]. As such, a vicious cycle of limitations in self-care and poor patient outcomes may develop [15]. This may further be exacerbated by a reduced ability to cope with the burden of multiple treatment regimens, increased risk of functional dependence and reduced chances of survival [15].

Establishing relationships between multimorbidity and function has clinical value, especially in primary care. Simple, low-cost assessments (such as walking speed or handgrip strength [17]) may for example be valid markers for clinical evaluation and monitoring in multimorbidity, and could be included in prevention or intervention strategies [17–20]. In a Cochrane review of community-based interventions to improve outcomes in multimorbidity care [21], significantly improved functional capacity was reported following occupational therapist- and physiotherapist-led interventions. Thus, it is important for healthcare providers to better understand the interplay of multimorbidity and functional status, considering its potential implications for patient management and outcomes.

### Multimorbidity patterns

Certain chronic conditions seem more likely to co-exist in associative patterns, possibly due to shared pathophysiological mechanisms or risk factors [22]. It is suggested that some multimorbid disease combinations have larger synergistic effects on health outcomes (including function and disability) and service utilization than others [4, 22, 23]. Multimorbidity has most often been described using simple or weighted disease counts [14]. Although count-based approaches are useful for identifying patients who require complex care [24], they are less helpful for informing clinical guidelines, as no distinction can be made between individuals with a similar amount, but different types, of diseases [11]. Statistical techniques are therefore increasingly used to categorize multimorbidity patterns into distinct non-random or associative classes. Although multimorbidity patterns vary depending on the analytical method used [25], systematic reviews on statistically determined profiles have described patterns of cardio-metabolic, mental health, and musculoskeletal problems with relative consistency [11, 22]; and it is suggested that replicable and clinically meaningful multimorbidity patterns do indeed exist [11].

Evidence on multimorbidity patterns in the specific context of LMICs, however, remains scarce; including information on patterns that may be associated with functional aspects (activity limitations, impairments of body function/structure and participation restriction). It has been suggested that cardio-respiratory, metabolic and mental health patterns are common regardless of country or income level [12, 22]. Other patterns that have been identified in low-resource settings include HIV and anemia [26], mental-articular [12], respiratory (including tuberculosis) [23], mental-sensory, and visceral-arthritic [23, 27].

Studies investigating multimorbidity and function have mostly focused on relationships in terms of the presence/absence of multimorbidity, or disease count, rather than multimorbidity patterns per se [27]. Yet, multimorbidity may have a different compound impact on function than the expected summed effect of single conditions. Diseases that co-exist in patterns may interact in complex ways, inhibiting compensatory mechanisms, which may lead to more severe functional problems [23, 28]. The severity of chronic diseases, order of onset, temporal evolution and social factors also add to the burden of multimorbidity on the individual [23]. In HICs, patterns of neuropsychiatric diseases have been shown to lead to the greatest declines in functional ability or instrumented activities of daily living (IADL) over time [29, 30], while cardiovascular profiles were associated only with declines in mobility [29] or activities of daily living (ADL) [30]. In LMIC studies, chronic lung disease and tuberculosis [23] and mental-sensory combinations (psychiatric conditions, cognition-related conditions, vision impairment, and hearing loss) [27] have been associated with the worst functional outcomes. Identifying multimorbidity patterns may provide insights on synergies and effects associated with coexisting conditions and aid recognition of more vulnerable patients that need special consideration when formulating care plans, secondary and tertiary prevention [24]. Therefore, identifying and understanding disease combinations that reliably present as patterns may contribute towards the development of guidelines that target specific profiles, risk factors and consequences [11]. Such guidelines may subsequently inform comprehensive service configurations to better address patient needs.

### Risk factors

Globally, multimorbidity is more reported among people of older age, male sex, and unemployed status; while seemingly less common in those with higher education levels and socioeconomic status [12, 26, 31]. Although on a global level, there seems to be agreement regarding common profiles of associated factors for multimorbidity, uncertainty remains regarding risk factors that predict specific multimorbidity patterns. This is largely due to lacking longitudinal evidence [4]. Additionally, although risk and/or protective factors for multimorbidity or poor function have been investigated separately, both health constructs have rarely been considered concurrently. Most studies of the relationship between multimorbidity and function have either been cross-sectional, or focused on multimorbidity as a one-directional predictor for functional decline incidence [15]. Data on the shared risk factors for multimorbidity and activity limitations of functional impairments thus remain limited [15], and non-existing in LMICs.

If multimorbidity leads to functional problems and/or vice versa, then the existence or development of one of these states may start a cascade effect, with a resulting escalation of risk for other adverse outcomes such as disability, decreased quality of life, and institutionalization [15]. Furthermore, if these phenomena have shared risk factors, then individuals with these characteristics would be at particularly high risk of developing a vicious cycle [15], and this group would be important to target for prevention strategies to mitigate poor outcomes.

### Implications for patients and the health system in low- and middle-income countries

Many LMICs continue to battle prevalent infectious diseases in parallel with high rates of non-communicable diseases, and will have to navigate the consequent population health, health systems, and economic implications [2, 32]. In South Africa, for example, multimorbid infectious and non-communicable diseases are the commonest in 40- to 60-year-olds [33]. People of these ages are usually engaged in employment and/or have domestic, family, and social responsibilities. Not only does multimorbidity have potential profoundly negative effects on health and work productivity, but the utilization and cost of care increase exponentially with the number of coexisting chronic conditions [34]. The burden on already resource-constrained health care systems is thus increased [35]; even more so if management of associated impairments will be needed at earlier ages and for longer periods.

Unfortunately, most health care systems, including those in LMICs, are structured to provide care in a vertical, disease-specific and curative nature [36, 37], rather than to provide organized care for chronic conditions. Such curative approaches are often inadequate, inefficient, and ineffective when a multiplex of chronic conditions coexist [38]. This is partly reflected in the observation that high levels of unmet treatment needs persist among people with multimorbidity [39].

When healthcare is focused on comprehensive guidelines-based treatment for a single condition (that may have led to a significant event such as hospitalization), concurrent conditions that have a similar or larger impact on the patient’s overall health may be missed [40]. In such scenarios, less (if any) attention may for instance be paid to managing functional problems—for example rising from a chair. Such specific functional problems may be very important to the person, and may indeed lead to future readmissions and/or prolonged hospitalization [41]. This affirms the need for person-centered, rather than disease-centered, care for people with multimorbidity throughout the various levels of care within the health system. To ensure that health and rehabilitation services are person-centered and health systems are responsive to rapidly evolving health care demands, there is an urgent need for health systems in LMICs to transit from specialized towards integrated health management models. This would be realized most successfully only if guided by research into multimorbidity epidemiological patterns, associated factors, function, and function-related impact.

### Rationale

The global move towards primary care led chronic disease management [42] has highlighted the identification of multimorbidity patterns and severity (for example measured by functional impairment, activity limitations and/or participation restrictions) as a research priority, given its correlation with patient outcomes [4, 23]. Most countries, including LMICs, are advocating for and making deliberate efforts towards the realization of Universal Health Coverage (UHC). The intention behind this move is ensuring access to quality and effective health services, without undue financial hardship. In LMICs, the goal of UHC may unfortunately be hard to realize for patients with multimorbidity, as the health systems are not equipped to deal with complex chronic conditions [35]. The management of patients with multimorbidity provide significant challenges to health- and rehabilitation specialists. Existing clinical guidelines for multimorbidity management [43–45] hail from HICs, making application in LMIC contexts inappropriate. Furthermore, these guidelines mostly use count-based definitions of multimorbidity. This is problematic, as the same number but different types of chronic diseases will likely have different risk profiles, treatment needs, and outcomes [11].

Appropriate evidence is needed to inform the development of locally relevant person-centered practice guidelines tailored to the needs of people with specific multimorbidity patterns. Of particular interest would be evidence about patterns of chronic conditions that tend to cluster together in low-resource contexts, and have the greatest impact on person-important outcomes such as function. Additionally, there is a need to tease out modifiable risk factors and pathways that may be common to multimorbidity and functional status. Ultimately, improved understanding of the needs and functional outcomes of individuals with different multimorbidity patterns will enable the transition from disease-centered to person-centered care for those living and aging with multimorbidity [11]. As quality of care would improve and the financial burden associated with multimorbidity and functional decline likely lessen, this would be an important step towards attaining UHC in LMICs.

### Aim of the planned scoping review

The aim of the planned scoping review is to provide an overview of the scope and nature of the existing literature on associations between multimorbidity patterns and function in adults, specifically in the context of LMICs. We plan to conduct a systematic search of peer-reviewed literature to scope the field, map the study characteristics, themes, and methodologies used in existing literature and to subsequently identify limitations and evidence gaps. By doing so we hope to provide recommendations to guide future research on multimorbidity patterns and functional associations in the context of low-resource countries.

## Methods

### Review design and framework

The study will be a comprehensive scoping review and will follow a five-step methodological framework adapted from the guidelines developed by Arksey and O’Malley [46] and refined by Levac et al. [47] and Peters et al. [48, 49]. The steps that will guide the review process are: (1) identifying the research question(s); (2) identifying relevant studies; (3) selecting the studies; (4) charting the data; and (5) collating, summarizing and reporting the results. The scoping review will be reported in accordance with the Preferred Reporting Items for Systematic Reviews and Meta-Analyses extension for Scoping Reviews (PRISMA-ScR) guidelines [50] (see Additional file 2 for the populated PRISMA for review protocols [PRISMA-P] checklist for the current protocol).

As a framework to assess and describe function, the International Classification of Functioning, Disability and Health (ICF) [51], will be used to code functional problems in terms of physical functional impairments, activity limitations and participation restrictions [52] (see Additional file 1 for definitions). Using the ICF as a framework will enable systematic coding and synthesis of concepts relevant to functional impairments and activity limitations derived from different outcome measures, disciplines, geographical and health care settings, and publication timeframes.

#### Step 1: Defining the research question(s)

This initial step will provide a roadmap for the review to guide the scoping process. The main research question for this review will be *“What is the scope and nature of existing literature on multimorbidity patterns that are associated with functional impairments and/or activity limitations and/or participation restrictions among adults in LMICs?”* This research question will allow us to gauge the full breadth of existing literature, while at the same time providing guidance towards potential syntheses should sufficient homogeneous constructs be identified, and the development of additional research questions. The main constructs of the research question were defined according to the Population, Exposure, Context and Outcome (PECO) framework [49] (Table 1).

**Table 1:** The PECO question framework for the proposed scoping review

Population	Exposure/condition^a^	Context	Outcome^a^
Adults (aged 18 years and older).	Multimorbidity patterns.	Low- and middle-income countries (LMICs), i.e. low-income, lower-middle-income and upper-middle-income countries as classified by the World Bank [53].	Activity limitations, impairments of body function and participation restrictions.

^a^ The concepts of multimorbidity and activity limitations/impairments/participation restrictions will be considered either the main outcome or exposures of interest, considering the potential bidirectionality of multimorbidity–function relationships

Accordingly, the following objectives will be addressed:

1. To describe what types of studies have been conducted on multimorbidity patterns and associated functional problems (i.e., impairments and/or activity limitations and/or participation restrictions) among adults in LMICs;
2. To describe in which countries and settings studies on multimorbidity patterns and associated functional problems among adults in LMICs have been conducted;
3. To describe the target populations that were included in studies on multimorbidity patterns and associated functional problems among adults in LMICs;
4. To describe how multimorbidity patterns or profiles were defined and determined (including statistical methods used) in studies on multimorbidity patterns and associated functional problems among adults in LMICs;
  - As a secondary objective, describe the clusters that were identified;
5. To describe how physical functional problems were assessed (which tool[s] were used);
  - As a secondary objective, describe the specific impairments and/or activity limitations and/or participation restrictions that were identified, and what the relationships to specific clusters were;
6. To describe any socio-demographic, lifestyle or other associated or risk factors reported for specific multimorbidity patterns and/or functional problems;
7. To identify similarities across studies in methodologies used for the determination and descriptions of multimorbidity patterns and functional problems;
8. To identify gaps in the literature and areas for future research (including syntheses) that would aid better understanding of multimorbidity patterns and associated functional problems among adults in LMICs.

#### Step 2: Identifying relevant studies

A comprehensive search will be conducted in consultation with the liaison librarian at Stellenbosch University. The following electronic databases will be searched: PubMed/MEDLINE, Scopus, EBSCOhost (including the Cumulative Index to Nursing and Allied Health Literature [CINAHL]), Scielo, Cochrane, and Google Scholar. Studies will be identified using search syntaxes based on a strategy developed in PubMed, which will use a combination of MeSH and free-text terms related to multimorbidity, clustering patterns, function and LMICs. The pilot syntax will initially also include keywords related to comorbidity, given the historical inconsistent use of terminology related to multimorbidity [4]. Additional file 3 lists the proposed keywords, which we adapted from other systematic reviews [11, 22], the Cochrane “LMIC Databases” document [54] and pilot searches ran on 02 September 2020. The final search syntax will subsequently be adapted for each database, including different or additional search terms as needed.

We will search in an iterative manner, including replacement of the names of different countries that constitute LMICs, and which are known to have people actively involved in multimorbidity research. The search will be limited to humans, adults, publication date (see “Eligibility criteria” section below) and language (English or Afrikaans). Search results will be imported into a reference management software package such as Mendeley and any duplicates in the database will be eliminated prior to screening studies for inclusion. In addition, the reference lists of included studies, as well as the International Research Community on Multimorbidity (IRCMo) library [55], will be searched manually to identify potentially eligible studies that might not have been identified by the primary search approach.

#### Step 3: Study selection (screening)

Following deduplication, articles will be screened for inclusion. Two reviewers (NT and KB) will independently review the initial hits from the comprehensive searches and apply the below-listed eligibility criteria to all titles and abstracts. Screened titles and abstracts will be categorized as “include”, “exclude” or “uncertain”.

Full texts of “included” and “uncertain” titles/abstracts (as classified by at least one reviewer) will subsequently be retrieved and assessed independently by the two reviewers against the eligibility criteria. Discrepancies will be discussed between the two reviewers, and the third reviewer will be consulted if needed. Studies that are non-eligible will be excluded from the review and reasons for exclusion will be recorded. The selection process will be summarized using a PRISMA-ScR flow diagram.

##### Eligibility criteria

For inclusion in the review, studies will have to meet the following criteria:

***Types of studies:***
1. Studies must be peer-reviewed, published full-text articles or conference proceedings of any design (primary or secondary; quantitative or qualitative).
2. Studies must have been published between January 1976 and the date of the final search. The date limit criterion was set given that the concept of “multimorbidity” first appeared in the literature in 1976 [56, 57] and since constant improvements in clinical care, public health and technology may affect rates of poor function and disability [58].
3. Studies must be available in English or Afrikaans.

***Types of participants:***
4. Studies must include adults (18 years and older) with multimorbidity living in a LMIC. If a study is found to also include individuals younger than 18, we will only include the study if separate results are reported for those 18 and older.
5. Studies will be excluded if they selected populations based on the presence of a pre-defined disease (i.e., studies that comorbidity in samples with an “index disease” or disease of main interest for study/treatment purposes). An example would be a study describing other diseases noted in a sample of adults with diabetes. This criterion will ensure that we only include studies on multimorbidity as operationally defined in this review.

***Types of outcome measures:***
6. Studies must refer to associations between multimorbidity patterns of co-occurrence (i.e., not only use disease counts) and physical function. The concept of multimorbidity must be addressed as operationally defined in this review (see Additional file 1). Studies will be excluded from the review if they define multimorbidity as the existence of co-morbidities in association with an index condition [4].
7. We will include studies regardless of the minimum number of chronic health conditions included in their analyses of multimorbidity patterns. Although previous systematic reviews [11, 22] have recommended that only studies using a minimum of 10 conditions when defining patterns should be included, we will not screen studies based on this criterion given (i) the expected paucity of literature, and (ii) that the purpose of this review is to provide a broad overview of methods used. We will, however, record how many conditions studies included in their analyses as part of our data extraction.
8. For these same reasons mentioned above, we will include studies that did *not* use statistical techniques to identify patterns (e.g., those describing frequencies of disease combinations), along with studies that *did* use statistical methods to determine patterns of co-occurrence.
9. Functional outcome measures may include subjective and objective measures of physical functional impairments, activity limitations or participation restrictions. Examples include impairment-based measures (e.g., handgrip strength measurement using dynamometry), patient-reported outcome measures (e.g., the Short Form 36 Health Survey Questionnaire), and/or physical performance-based tests (e.g., the five-times sit-to-stand test).

#### Step 4: Data charting

The full review team have discussed the information to be extracted from the included studies and have developed a pilot data charting form in Microsoft Excel (Additional file 4). This preliminary data extraction form will be piloted independently by two of the reviewers (KB and NT) on four (if possible; depending on number of eligible articles identified) randomly selected studies. The form will be modified iteratively based on experiences of extracting data from each of the four articles, and before commencing data extraction on the full list of included articles. This will ensure appropriateness and consistency of the data extraction process going forward. Final data extraction will be done independently by two reviewers (NT and KB) and any disagreements will be resolved by discussion. If consensus is not achieved, the third reviewer (QL) will be consulted.

It is envisaged that the final form will include information on the following: *study characteristics* (first author, publication year, study design, study aim/objectives/questions, country, setting, sample size, limitations, main findings/conclusions); *participant characteristics* (target population, age, sex); *multimorbidity measures* (multimorbidity definition, method of ascertaining chronic conditions, diagnosis classification tool, disease inclusion criteria), *pattern analysis* (method or statistical technique used to determine patterns, number of diseases/conditions analyzed, disease grouping algorithm, use of stratification, criteria to determine number of patterns extracted); *functional measures* (definition of function, outcome measure/tool used); *results* (multimorbidity prevalence, functional impairment/activity limitation/participation restriction prevalence, number of multimorbidity patterns identified, allocated names of patterns, diseases included in each pattern, function-related symptom associated with each pattern, description of multimorbidity-function relationship for each pattern, other associated/risk factors for each pattern); and *ICF-related codes of functional outcomes associated with each pattern* (ICF component, ICF domain/chapter, ICF category, and ICF qualifier). We will also add a column for qualitative data, and to document reasons for exclusion, should a study be found ineligible for inclusion in the review at the stage of full-text screening.

#### Step 5: Evidence synthesis and reporting of results

The extracted data will be collated, summarized, and reported in a manner that maps the breadth of existing published literature in the field of multimorbidity patterns and function in the context of LMICs, with a focus on *how* research in this field has been conducted. As such, we will establish the methodologies and assessments that have been used to determine multimorbidity patterns and functional problems, and whether methods and results are comparable across studies. One of the envisaged outcomes of such an aggregate overview is establishing the feasibility of a more focused future review and synthesis. All data generated, analyzed, and reported for the purposes of this review will be included in the published scoping review article, which will be reported according to the PRISMA-ScR guidelines. Any amendments to the current protocol will be documented and published with the final review report.

The review results will determine the precise reporting format and products; however, we envisage to present results in terms of search results, study and participant characteristics, methodological aspects of determining and describing multimorbidity patterns and function, relationships between multimorbidity patterns and functional problems, and additional factors associated with either. As a variety of study designs will likely be found among included studies, we will use descriptive statistics and narrative synthesis of extracted information. Data will be presented as tables, charts, and visual maps (including a PRISMA-ScR flow diagram). The activity limitations, impairments, and/or participation restrictions identified in the included studies will be coded by two reviewers (NT and KB) using the ICF framework. Should impairments or activity limitations not be explicitly reported, the main concepts from the outcome measures used to evaluate physical function will be used to derive the functional impairment or activity limitation using ICF linking rules.

### Anticipated challenges

The quantity of studies identified (either too many or too little) for potential inclusion may pose a threat. We may thus need to either refine or broaden our review focus to ensure it is feasible; such amendments may be considered within the review team after the comprehensive search (which will be performed and documented as set out in the methods) but before title/abstract screening and charting phases. We will also consider writing an empty review, should the comprehensive search render no studies on the research topic. We anticipate varying definitions of multimorbidity, varying methods to determine clustering patterns, the use of non-standardized testing/tools, cross-country differences regarding coding systems, and factors such as poor development and limited access to health services, which may limit syntheses of findings beyond describing what has been done in the field. Such limitations, and the implication thereof for interpretation of the results, will be explicitly stated in the published scoping review.

## Discussion

Multimorbidity is becoming an increasing concern for already-constrained healthcare systems in LMICs due to its rising prevalence, complex management, economic burden, and negative association with patient outcomes. LMICs face complex, multiple disease burdens among relatively younger, yet aging, populations [59]. This implies that multimorbidity patterns and functional sequelae will likely differ from those in HICs. As LMICs aspire to achieve UHC, there is a growing need for improved clinical outcomes and quality of care, which highlights the importance of person-centered—as opposed to disease-centered—care. However, owing to the vertical, curative orientation of healthcare systems in LMICs, and the disease-specific focus of existing clinical guidelines, the delivery of healthcare remains siloed to single-diseases. This issue includes rehabilitation, which is integral to chronic disease management. People with multimorbidity are thus managed using multiple single-disease guidelines; even though single-disease care may be inappropriate.

High levels of treatment dissatisfaction [39] and prevalent functional problems, functional decline and disability [3, 6] remain among people with multimorbidity. Recently, the Academy of Medical Sciences identified clarity about multimorbidity patterns, and associated burden (including on person-centric outcomes such as function) of specific patterns in specific contexts, as research priorities [4]. Increased clarity in this regard will help to inform healthcare policy and investment decisions [4]. Research on typical disease combinations and associated functional problems within the ambit of multimorbidity in LMICs seems to be emerging, but the evidence remains scanty, fragmented, and poorly understood [4, 5, 11]. However, evidence from HICs, and emergingly from LMICs, suggests that different combinations of chronic diseases are differentially associated with poor functional outcomes [4, 22, 23]. As the adverse outcomes associated with multimorbidity are largely attributable to the patterning and types of co-occurring conditions, patterns—rather than disease count—are of importance to inform appropriate clinical management and understanding of the disease effect by researchers and policy-makers [23]. Particularly, more evidence regarding the existence and impact of typical multimorbidity patterns could inform decision-making about resource allocation, service provision and management strategies to ensure people with multimorbidity receive the best possible care.

With the shift towards primary healthcare-led management of chronic diseases and UHC, the proposed scoping review is timely. The review will aim to map existing literature on associations between multimorbidity patterns and function in adults, specifically in the context of LMICs. Review results will provide the first insights into the extent and scope of multimorbidity research in this field, and will expose similarities and differences in methodologies, along with existing gaps in the literature, to guide future inquiry in the field. Understanding relationships between specific multimorbidity patterns and physical function and may aid prevention of a cascade toward poorer health outcomes. In the longer term, such understanding will contribute towards informing the development of evidence-based and context-specific health policy, service planning and delivery programs. This will be in tandem with the ongoing shift from specialized to integrated health care systems, and from disease-centered to person-centered health and rehabilitative care for people living with multimorbidity in LMICs.

## Supplementary Information

**Additional file 1.** Glossary of key terms relevant to the scoping review protocol. A list of definitions of important concepts referred to in the main manuscript.

**Additional file 2.** PRISMA-P (Preferred Reporting Items for Systematic review and Meta-Analysis Protocols) 2015 checklist. Completed PRISMA-P Checklist for the review protocol, indicating where (as applicable at the stage of a protocol) relevant sections are addressed.

**Additional file 3.** Proposed keywords to be used in the search syntax. A list of proposed keywords for the pilot PubMed-based search strategy, including a list of low-income and middle-income countries as classified by the World Bank.

**Additional file 4.** Pilot data extraction form. Pilot data extraction categories developed for the scoping review.

## Data Availability

All data generated and analyzed in this review will be reported and published in a full review manuscript, which will be published in a peer-reviewed open-access journal. Data generated from this review will be made available on open access repository at Stellenbosch University. Datasets will be available from the lead author on request or from the open access Stellenbosch University repository.

## Abbreviations

HICs: High-income countries
LMICs: Low-income and middle-income countries
UHC: Universal Health Coverage
WHO: World Health Organization

## References

[1] Levitt NS, Steyn K, Dave J, Bradshaw D (2011). Chronic noncommunicable diseases and HIV-AIDS on a collision course: relevance for health care delivery, particularly in low-resource settings--insights from South Africa. Am J Clin Nutr.

[2] Lalkhen H, Mash R (2015). Multimorbidity in non-communicable diseases in South African primary healthcare. South Afr Med J.

[3] Arokiasamy P, Uttamacharya U, Jain K, Biritwum RB, Yawson AE, Wu F (2015). The impact of multimorbidity on adult physical and mental health in low- and middle-income countries: what does the study on global ageing and adult health (SAGE) reveal?. BMC Med.

[4] The Academy of Medical Sciences (2018). Multimorbidity: a priority for global research.

[5] Nguyen H, Manolova G, Daskalopoulou C, Vitoratou S, Prince M, Prina AM (2019). Prevalence of multimorbidity in community settings: a systematic review and meta-analysis of observational studies. J Comorbidity.

[6] Payne CF, Wade A, Kabudula CW, Davies JI, Chang AY, Gomez-Olive FX (2017). Prevalence and correlates of frailty in an older rural African population: findings from the HAALSI cohort study. BMC Geriatr.

[7] Arokiasamy P, Uttamacharya U, Jain K (2014). Multiple chronic diseases and their linkages with functional health and subjective wellbeing among adults in the low-middle income countries: an analysis of SAGE Wave1 Data, 2007/10.

[8] Lee JT, Hamid F, Pati S, Atun R, Millett C (2015). Impact of noncommunicable disease multimorbidity on healthcare utilisation and out-of-pocket expenditures in middle-income countries: cross sectional analysis. PLoS One.

[9] Barnett K, Mercer SW, Norbury M, Watt G, Wyke S, Guthrie B (2012). Epidemiology of multimorbidity and implications for health care, research, and medical education: a cross-sectional study. Lancet (London, England).

[10] Guthrie B, Payne K, Alderson P, McMurdo METT, Mercer SW (2012). Adapting clinical guidelines to take account of multimorbidity. BMJ..

[11] Busija L, Lim K, Szoeke C, Sanders KM, McCabe MP (2019). Do replicable profiles of multimorbidity exist? Systematic review and synthesis. Eur J Epidemiol.

[12] Garin N, Koyanagi A, Chatterji S, Tyrovolas S, Olaya B, Leonardi M (2016). Global Multimorbidity Patterns: A Cross-Sectional, Population-Based, Multi-Country Study. J Gerontol - Ser A Biol Sci Med Sci.

[13] Xu X, Mishra GD, Jones M (2017). Evidence on multimorbidity from definition to intervention: An overview of systematic reviews. Ageing Res Rev.

[14] De Francesco D, Sabin CA, Reiss P (2020). Multimorbidity patterns in people with HIV. Curr Opin HIV AIDS.

[15] Calderón-Larrañaga A, Vetrano DL, Ferrucci L, Mercer SW, Marengoni A, Onder G (2019). Multimorbidity and functional impairment-bidirectional interplay, synergistic effects and common pathways. J Intern Med.

[16] Ryan A, Wallace E, O’Hara P, Smith SM (2015). Multimorbidity and functional decline in community-dwelling adults: A systematic review. Health Qual Life Outcomes.

[17] Ryan A, Murphy C, Boland F, Galvin R, Smith SM (2018). What is the impact of physical activity and physical function on the development of multimorbidity in older adults over time? A population-based cohort study. Journals Gerontol - Ser A Biol Sci Med Sci.

[18] Ortiz PJ, Tello T, Aliaga EG, Casas PM, Peinado JE, Miranda JJ (2018). Effect of multimorbidity on gait speed in well-functioning older people: a population-based study in Peru. Geriatr Gerontol Int.

[19] de Amaral CA, Portela MC, Muniz PT, Dos Santos FE, de Araújo TS, de Souza OF (2015). Association of handgrip strength with self-reported diseases in adults in Rio Branco, Acre State, Brazil: a population-based study. Cad Saude Publica.

[20] Pessini J, Barbosa AR, Trindade EBSDM (2016). Chronic diseases, multimorbidity, and handgrip strength among older adults from Southern Brazil. Rev Nutr.

[21] Smith SM, Wallace E, O’Dowd T, Fortin M (2016). Interventions for improving outcomes in patients with multimorbidity in primary care and community settings. Cochrane Database Syst Rev.

[22] Prados-Torres A, Calderón-Larrañaga A, Hancco-Saavedra J, Poblador-Plou B, Van Den Akker M (2014). Multimorbidity patterns: a systematic review. J Clin Epidemiol.

[23] Pati S, Swain S, Metsemakers J, Knottnerus JA, van den Akker M (2017). Pattern and severity of multimorbidity among patients attending primary care settings in Odisha, India. PLoS One.

[24] Harrison C, Britt H, Miller G, Henderson J (2014). Examining different measures of multimorbidity, using a large prospective cross-sectional study in Australian general practice. BMJ Open.

[25] Roso-Llorach A, Violán C, Foguet-Boreu Q, Rodriguez-Blanco T, Pons-Vigués M, Pujol-Ribera E (2018). Comparative analysis of methods for identifying multimorbidity patterns: A study of “real-world” data. BMJ Open.

[26] Chang AY, Gómez-Olivé FX, Payne C, Rohr JK, Manne-Goehler J, Wade AN (2019). Chronic multimorbidity among older adults in rural South Africa. BMJ Glob Health.

[27] Yao SS, Meng X, Cao GY, Huang ZT, Chen ZS, Han L (2020). Associations between multimorbidity and physical performance in older Chinese adults. Int J Environ Res Public Health.

[28] Marengoni A, Angleman S, Fratiglioni L (2011). Prevalence of disability according to multimorbidity and disease clustering: a population-based study. J Comorbidity.

[29] Vetrano DL, Rizzuto D, Calderón-Larrañaga A, Onder G, Welmer AK, Bernabei R, et al. Trajectories of functional decline in older adults with neuropsychiatric and cardiovascular multimorbidity: A Swedish cohort study. PLoS Med. 2018;15. 10.1371/journal.pmed.1002503. 10.1371/journal.pmed.1002503 PMC5839531 29509768

[30] Jackson CA, Jones M, Tooth L, Mishra GD, Byles J, Dobson A (2015). Multimorbidity patterns are differentially associated with functional ability and decline in a longitudinal cohort of older women. Age Ageing.

[31] Weimann A, Dai D, Oni T (2016). A cross-sectional and spatial analysis of the prevalence of multimorbidity and its association with socioeconomic disadvantage in South Africa: a comparison between 2008 and 2012. Soc Sci Med.

[32] Oni T, Unwin N (2015). Why the communicable/non-communicable disease dichotomy is problematic for public health control strategies: Implications of multimorbidity for health systems in an era of health transition. Int Health.

[33] Sharman M, Bachmann M (2019). Prevalence and health effects of communicable and non-communicable disease comorbidity in rural KwaZulu-Natal, South Africa. Trop Med Int Health.

[34] Picco L, Achilla E, Abdin E, Chong SA, Vaingankar JA, McCrone P, et al. Economic burden of multimorbidity among older adults: Impact on healthcare and societal costs. BMC Health Serv Res. 2016;16. 10.1186/s12913-016-1421-7. 10.1186/s12913-016-1421-7 PMC4862090 27160080

[35] Valderas JM, Starfield B, Sibbald B, Salisbury C, Roland M (2009). Defining comorbidity: Implications for understanding health and health services. Ann Fam Med.

[36] Mahomed OH, Asmall S, Voce A (2016). Sustainability of the integrated chronic disease management model at primary care clinics in South Africa. Afr J Prim Heal Care Fam Med..

[37] Osakunor DNM, Sengeh DM, Mutapi F (2018). Coinfections and comorbidities in African health systems: At the interface of infectious and noninfectious diseases. PLoS Negl Trop Dis.

[38] Nelson MLA, McKellar KA, Yi J, Kelloway L, Munce S, Cott C (2017). Stroke rehabilitation evidence and comorbidity: a systematic scoping review of randomized controlled trials. Top Stroke Rehabil.

[39] Folb N, Timmerman V, Levitt NS, Steyn K, Bachmann MO, Lund C (2015). Multimorbidity, control and treatment of noncommunicable diseases among primary healthcare attenders in the Western Cape, South Africa. S Afr Med J.

[40] Zulman DM, Asch SM, Martins SB, Kerr EA, Hoffman BB, Goldstein MK (2014). Quality of care for patients with multiple chronic conditions: The role of comorbidity interrelatedness. J Gen Intern Med.

[41] Greysen SR, Cenzer IS, Auerbach AD, Covinsky KE (2015). Functional impairment and hospital readmission in medicare seniors. JAMA Intern Med.

[42] Reynolds R, Dennis S, Hasan I, Slewa J, Chen W, Tian D (2018). A systematic review of chronic disease management interventions in primary care. BMC Fam Pract.

[43] Palmer K, Marengoni A, Forjaz MJ, Jureviciene E, Laatikainen T, Mammarella F (2018). Multimorbidity care model: Recommendations from the consensus meeting of the Joint Action on Chronic Diseases and Promoting Healthy Ageing across the Life Cycle (JA-CHRODIS). Health Policy..

[44] National Institute for Health and Care Excellence. Multimorbidity: clinical assessment and management: NICE guideline [NG56]. London; 2016. https://www.nice.org.uk/guidance/ng56. Accessed 8 Oct 2020.

[45] US Department of Health and Human Services (2010). Multiple chronic conditions—a strategic framework: optimum health and quality of life for individuals with multiple chronic conditions.

[46] Arksey H, O’Malley L (2005). Scoping studies: towards a methodological framework. Int J Soc Res Methodol.

[47] Levac D, Colquhoun H, O’Brien KK (2010). Scoping studies: advancing the methodology. Implement Sci.

[48] Munn Z, Peters MDJ, Stern C, Tufanaru C, McArthur A, Aromataris E (2018). Systematic review or scoping review? Guidance for authors when choosing between a systematic or scoping review approach. BMC Med Res Methodol.

[49] Peters M, Godfrey C, McInerey P, Munn Z, Tricco A, Khalil H, Aromataris E, Munn Z (2020). Chapter 11: Scoping Reviews (2020 version). JBI Manual for Evidence Synthesis.

[50] Tricco AC, Lillie E, Zarin W, O’Brien KK, Colquhoun H, Levac D (2018). PRISMA extension for scoping reviews (PRISMA-ScR): Checklist and explanation. Ann Intern Med.

[51] World Health Organisation (2001). International Classification of Functioning, Disability and Health: ICF.

[52] Prodinger B, Stucki G, Coenen M, Tennant A (2019). The measurement of functioning using the International Classification of Functioning, Disability and Health: comparing qualifier ratings with existing health status instruments. Disabil Rehabil.

[53] World Bank Group (2020). World Bank Country and Lending Groups.

[54] Cochrane (2013). LMIC Databases. A collection of databases, web sites and journals relevant toLow-and Middle-Income Countries (LMIC).

[55] Fortin M (2016). IRCMo. International Research Community on Multimorbidity.

[56] Brandlmeier P (1976). Multimorbidity among elderly patients in an urban general practice. Z Allgemeinmed.

[57] Le Reste JY, Nabbe P, Rivet C, Lygidakis C, Doerr C, Czachowski S (2015). The European general practice research network presents the translations of its comprehensive definition of multimorbidity in family medicine in ten European languages. PLoS One.

[58] Groce NE (2018). Global disability: an emerging issue. Lancet Glob Health.

[59] Hurst JR, Agarwal G, Van Boven JFM, Daivadanam M, Gould GS, Wan-Chun Huang E, et al. Critical review of multimorbidity outcome measures suitable for low-income and middle-income country settings: Perspectives from the Global Alliance for Chronic Diseases (GACD) researchers. BMJ Open. 2020;10. 10.1136/bmjopen-2020-037079. 10.1136/bmjopen-2020-037079 PMC7478040 32895277

