# Supplementary material for: Multimorbidity patterns and function among adults in low- and middle-income countries: a scoping review protocol
Source: Syst Rev. 2022 Jul 7;11:139. doi: 10.1186/s13643-022-01996-3 (PMC9261061; doi:10.1186/s13643-022-01996-3)
Supplement: Supplementary file 1 — Additional file 1. Glossary of key terms relevant to the scoping review protocol. A list of definitions of important concepts referred to in the main manuscript. [file 13643_2022_1996_MOESM1_ESM.docx]

**Additional file 1: Glossary of key terms relevant to the scoping review protocol**

**Activity limitation:** Defined in terms of the International Classification of Functioning, Disability and Health (ICF) [1, 2] as having difficulty in executing a task or action, such as having difficulty with walking or moving from sitting to standing. As “activity” refers to task execution by an individual, it denotes the individual perspective of functioning [3].

**Clinical Practice Guidelines:** Defined as “statements that include recommendations intended to optimize patient care that are informed by a systematic review of evidence and an assessment of the benefits and harms of alternative care options” [4].

**Co-morbidity:** The co-existence of other diseases in addition to an index disease (that is considered the main focus of attention/treatment) in an individual [5].

**Disability:** defined according to the ICF as the “negative aspects of interaction between a person with a health condition and that person’s contextual factors. It is an umbrella term for impairments of body function and structures, activity limitations and participation restrictions” [6].

**Functional problems:** For the planned scoping review, we will use the term “functional problems” as referring to the components of disability affecting what a person is (un)able to do at the level of the individual (as opposed to, for example, acceptance and integration from society’s part): including impairments of body function/structure, activity limitations and participation restrictions at person-level [3, 6].

**Health System:** All activities with the primary purpose of promoting, restoring and/or maintaining health; the people, institutions and resources, grouped together according to established policies, using a variety of activities to improve the health of the population they serve [7].

**Impairment:** Defined in terms of the ICF [1, 2] as a loss or abnormality in an anatomical structure (e.g. muscle function deficits or impaired joint mobility) or physiological function of body systems. Such impairments, individually or combined, may contribute to activity limitations and participation restrictions, and eventually a trajectory towards functional decline and disability [8].

**International Classification of Functioning, Disability and Health (ICF):** The World Health Organization (WHO)’s framework for health and disability. The ICF provides a universal, standardized and etiologically neutral way of describing health-related function [2].

**Low-income and middle-income countries (LMICs) and high-income countries (HICs):** For the 2021 fiscal year, the World Bank defines low-income countries as those with a gross national income (GNI) per capita of USD 1,035 or less in 2019; lower middle-income countries as those with a GNI per capita of USD 1,036 to 4,045; upper middle-income countries as those with a GNI per capita of USD 4,046 to 12,535. High-income countries are defined as those with a GNI per capita of USD 12,536 or more [9].

**Multimorbidity patterns:** Conditions that are more likely to co-exist or cluster together (either concordantly or discordantly), possibly due to shared biological or environmental factors, or pathological pathways where one condition may heighten the risk of another. Different clustering patterns of seem to occur in different geographical locations, as well as in different demographic groups [5].

**Multimorbidity:** Defined according to the Academy of Medical Sciences [5] and WHO [10] as the co-existence of two or more chronic diseases in an individual. The conditions may include any combination of physical non-communicable, infectious, and mental health conditions and may or may not interact with each other (e.g. regarding pathophysiology, clinical management, or patient impact). The concept of multimorbidity is distinguished from comorbidity in that none of the co-existing conditions are considered an index condition that is the specific focus of attention.

**Participation restriction:** Defined in terms of the ICF [1, 2] as problems an individual may have in participating in life situations. Examples include restrictions in going to work, social and recreational engagement, and accessing health care and preventive services; but – as Stucki et al. [3] points out – also in walking, if walking is integral to participation in terms of a life situation. As such, “participation (restriction)” denotes the social aspect of functioning [3].

**Risk factor:** Defined as an attribute, characteristic or exposure that is causally associated with an increased probability of developing a disease or injury. Examples include tobacco and alcohol use, and obesity [11, 12].

**Universal Health Coverage (UHC):** A healthcare system in which “all people and communities can use the promotive, preventive, curative, rehabilitative and palliative health services they need, of sufficient quality to be effective, while also ensuring that the use of these services does not expose the user to financial hardship” [13].

**References**

1. World Health Organisation. International Classification of Functioning, Disability and Health: ICF. Geneva: World Health Organization; 2001.

2. Prodinger B, Stucki G, Coenen M, Tennant A. The measurement of functioning using the International Classification of Functioning, Disability and Health: comparing qualifier ratings with existing health status instruments. Disabil Rehabil. 2019;41:541–8.

3. Stucki G, Melvin J. The international classification of functioning and disability and health: a unifying model for the conceptual description of physical and rehabilitative medicine. J Rehabil Med. 2007;39:286–92.

4. Clinical Practice Guideline Manual. https://www.aafp.org/family-physician/patient-care/clinical-recommendations/cpg-manual.html. Accessed 27 Oct 2020.

5. The Academy of Medical Sciences. Multimorbidity: a priority for global research. 2018.

6. WHO. How to use the ICF: A Practical Manual for using the International Classification of Functioning, Disability and Health (ICF). Exposure draft for comment. Geneva; 2013.

7. World Health Organization. Health Systems Strengthening Glossary. https://www.who.int/healthsystems/hss_glossary/en/index5.html. Accessed 27 Oct 2020.

8. Jette AM. Physical disablement concepts for physical therapy research and practice. Physical Therapy. 1994;74:380–6. doi:10.1093/ptj/74.5.380.

9. World Bank Group. World Bank Country and Lending Groups. 2020. https://datahelpdesk.worldbank.org/knowledgebase/articles/906519-world-bank-country-and-lending-groups. Accessed 22 Oct 2020.

10. World Health Organization. Multimorbidity: Technical Series on Safer Primary Care. Geneva; 2016.

11. OECD iLibrary | Health risks. https://www.oecd-ilibrary.org/social-issues-migration-health/health-risks/indicator-group/english_1c4df204-en. Accessed 27 Oct 2020.

12. Lopez A, Mahers C, Ezzati M, Jamison D, Murray CJ, editors. Global Burden of Disease and Risk Factors. Washington (DC): The International Bank for Reconstruction and Development / The World Bank; New York: Oxford University Press; 2006.

13. WHO | What is universal coverage? WHO. 2019. http://www.who.int/health_financing/universal_coverage_definition/en/. Accessed 27 Oct 2020.
