# Supplementary material for: Multimorbidity patterns and function among adults in low- and middle-income countries: a scoping review protocol
Source: Syst Rev. 2022 Jul 7;11:139. doi: 10.1186/s13643-022-01996-3 (PMC9261061; doi:10.1186/s13643-022-01996-3)
Supplement: Supplementary file 3 — Additional file 3. Proposed keywords to be used in the search syntax. A list of proposed keywords for the pilot PubMed-based search strategy, including a list of low-income and middle-income countries as classified by the World Bank. [file 13643_2022_1996_MOESM3_ESM.docx]

**Additional file 3: Proposed keywords to be used in the search syntax.**

**Supplementary Table A.** Proposed keywords to be used in the search syntax.

| **Multimorbidity** | To be combined using AND | **Patterns** | To be combined using AND | **Functional impairments, activity limitations or participation restrictions** | To be combined using AND | **Low- and middle-income countries (LMIC)** |
| --- | --- | --- | --- | --- | --- | --- |
| To be combined using OR |  | To be combined using OR |  | To be combined using OR |  | To be combined using OR |
| multimorbid*  “multimorbid* pattern*”  “multimorbid* cluster*”  “disease pattern*”  “disease cluster*”  “associative multimorbid*”  comorbidit*  polymorbidit*  polypatholog*  multipatholog*  multicondition*  "multiple comorbid*"  “multiple chronic condition*" |  | pattern*  cluster*  associative  “cluster analys*”  "factor analys*"  "principal component*" "correspondence analys*"  "multicorrespondence analys*"  "multi-correspondence analys*"  "latent class analys*"  "log-linear analys*"  "log linear analys*"  "loglinear analys*"  "multiway frequenc*"  "multi-way frequenc*" |  | function*  impairment*  impair*  participation  disabilit*  dysfunction*  capabilit*  mobilit*  movement*  ICF  “function* impairment*”  “function* deficit*”  “function* loss”  “activity limitation*”  “activity restriction*”  “participation restriction”  “functional decline”  “physical fitness”  “functional abilit*”  “functional capa*”  “functional status”  “functional assessment”  “functional performance”  “physical function”  “physical performance”  “international classification functioning disabil* health”  “classification functioning”  “classification impairment” |  | lmic*  “low* income countr*”  “low* income nation*”  “low* income population*”  “low* income world”  “low* income econom*”  “middle income countr*”  “middle income nation*”  “middle income population*”  “middle income world”  “middle income econom*”  “low* middle income countr*”  “low* middle income nation*”  “low* middle income population*”  “low* middle income world”  “low* middle income econom*”  “upper middle income countr*”  “upper middle income nation*”  “upper middle income population*”  “upper middle income world”  “upper middle income econom*”  “high middle income countr*”  “high middle income nation*”  “high middle income population*”  “high middle income world”  “high middle income econom*”  “developing countr*”  "developing nation*”  “developing population*”  “developing world”  “developing econom*”  “third world countr*”  “third world nation*”  “third world population*”  “third world econom*”  “transitional countr*”  “transitional nation*”  “transitional population*”  “transitional econom*”  “less* developed country”  “less* developed nation*”  “less* developed population*”  “less* developed world”  “less* developed econom*”  “under developed country”  “under developed nation*”  “under developed population*”  “under developed world”  “under developed econom*”  “underserved countr*”  “underserved nation*”  “underserved population*”  “underserved world”  “underserved econom*”  “deprived countr*”  “deprived nation*”  “deprived population*”  “deprived world”  “deprived econom*”  “poor* countr*”  “poor* nation*”  “poor* population*”  “poor* world”  “poor* econom*”  “low* gdp”  “low* gnp”  “low* gross domestic”  “low* gross national”  We will additionally include keywords as per the Cochrane “LMIC Databases” document [1] (e.g. as suggested for MEDLINE and PubMed), replacing the 2013 list of LMIC country names with the most recent World Bank Classification list [2] (see Supplementary Table B below for 2020 list). |

Spelling variations will be included, e.g. use of hyphens.
* indicates an open ending to the word.

**Supplementary Table B.** World Bank list of low-income and middle-income countries (LMIC), 2020.

| **Country** | **Income group** |
| --- | --- |
| Afghanistan | Low income |
| Albania | Upper middle income |
| Algeria | Lower middle income |
| American Samoa | Upper middle income |
| Angola | Lower middle income |
| Argentina | Upper middle income |
| Armenia | Upper middle income |
| Azerbaijan | Upper middle income |
| Bangladesh | Lower middle income |
| Belarus | Upper middle income |
| Belize | Upper middle income |
| Benin | Lower middle income |
| Bhutan | Lower middle income |
| Bolivia | Lower middle income |
| Bosnia and Herzegovina | Upper middle income |
| Botswana | Upper middle income |
| Brazil | Upper middle income |
| Bulgaria | Upper middle income |
| Burkina Faso | Low income |
| Burundi | Low income |
| Cabo Verde | Lower middle income |
| Cambodia | Lower middle income |
| Cameroon | Lower middle income |
| Central African Republic | Low income |
| Chad | Low income |
| China | Upper middle income |
| Colombia | Upper middle income |
| Comoros | Lower middle income |
| Congo, Dem. Rep. | Low income |
| Congo, Rep. | Lower middle income |
| Côte d'Ivoire | Lower middle income |
| Cuba | Upper middle income |
| Djibouti | Lower middle income |
| Dominica | Upper middle income |
| Dominican Republic | Upper middle income |
| Ecuador | Upper middle income |
| Egypt, Arab Rep. | Lower middle income |
| El Salvador | Lower middle income |
| Equatorial Guinea | Upper middle income |
| Eritrea | Low income |
| Eswatini | Lower middle income |
| Ethiopia | Low income |
| Fiji | Upper middle income |
| Gabon | Upper middle income |
| Gambia, The | Low income |
| Georgia | Upper middle income |
| Ghana | Lower middle income |
| Grenada | Upper middle income |
| Guatemala | Upper middle income |
| Guinea | Low income |
| Guinea-Bissau | Low income |
| Guyana | Upper middle income |
| Haiti | Low income |
| Honduras | Lower middle income |
| India | Lower middle income |
| Indonesia | Upper middle income |
| Iran, Islamic Rep. | Upper middle income |
| Iraq | Upper middle income |
| Jamaica | Upper middle income |
| Jordan | Upper middle income |
| Kazakhstan | Upper middle income |
| Kenya | Lower middle income |
| Kiribati | Lower middle income |
| Korea, Dem. People's Rep. | Low income |
| Kosovo | Upper middle income |
| Kyrgyz Republic | Lower middle income |
| Lao PDR | Lower middle income |
| Lebanon | Upper middle income |
| Lesotho | Lower middle income |
| Liberia | Low income |
| Libya | Upper middle income |
| Madagascar | Low income |
| Malawi | Low income |
| Malaysia | Upper middle income |
| Maldives | Upper middle income |
| Mali | Low income |
| Marshall Islands | Upper middle income |
| Mauritania | Lower middle income |
| Mexico | Upper middle income |
| Micronesia, Fed. Sts. | Lower middle income |
| Moldova | Lower middle income |
| Mongolia | Lower middle income |
| Montenegro | Upper middle income |
| Morocco | Lower middle income |
| Mozambique | Low income |
| Myanmar | Lower middle income |
| Namibia | Upper middle income |
| Nepal | Lower middle income |
| Nicaragua | Lower middle income |
| Niger | Low income |
| Nigeria | Lower middle income |
| North Macedonia | Upper middle income |
| Pakistan | Lower middle income |
| Papua New Guinea | Lower middle income |
| Paraguay | Upper middle income |
| Peru | Upper middle income |
| Philippines | Lower middle income |
| Russian Federation | Upper middle income |
| Rwanda | Low income |
| Samoa | Upper middle income |
| São Tomé and Principe | Lower middle income |
| Senegal | Lower middle income |
| Serbia | Upper middle income |
| Sierra Leone | Low income |
| Solomon Islands | Lower middle income |
| Somalia | Low income |
| South Africa | Upper middle income |
| South Sudan | Low income |
| Sri Lanka | Lower middle income |
| St. Lucia | Upper middle income |
| St. Vincent and the Grenadines | Upper middle income |
| Sudan | Low income |
| Suriname | Upper middle income |
| Syrian Arab Republic | Low income |
| Tajikistan | Low income |
| Tanzania | Lower middle income |
| Thailand | Upper middle income |
| Timor-Leste | Lower middle income |
| Togo | Low income |
| Tonga | Upper middle income |
| Tunisia | Lower middle income |
| Turkey | Upper middle income |
| Turkmenistan | Upper middle income |
| Tuvalu | Upper middle income |
| Uganda | Low income |
| Ukraine | Lower middle income |
| Uzbekistan | Lower middle income |
| Vanuatu | Lower middle income |
| Venezuela, RB | Upper middle income |
| Vietnam | Lower middle income |
| West Bank and Gaza | Lower middle income |
| Yemen, Rep. | Low income |
| Zambia | Lower middle income |
| Zimbabwe | Lower middle income |

**References**

1. Cochrane. LMIC Databases. A collection of databases,web sites and journals relevant toLow-and Middle-Income Countries (LMIC). 2013. https://epoc.cochrane.org/sites/epoc.cochrane.org/files/public/uploads/LMIC Databases August 2013.pdf. Accessed 23 Oct 2020.

2. World Bank Group. World Bank Country and Lending Groups. 2020. https://datahelpdesk.worldbank.org/knowledgebase/articles/906519-world-bank-country-and-lending-groups. Accessed 22 Oct 2020.
